# Patch-clamp recordings of rat neurons from acute brain slices of the somatosensory cortex during magnetic stimulation

**DOI:** 10.3389/fncel.2014.00145

**Published:** 2014-06-03

**Authors:** Tamar Pashut, Dafna Magidov, Hana Ben-Porat, Shuki Wolfus, Alex Friedman, Eli Perel, Michal Lavidor, Izhar Bar-Gad, Yosef Yeshurun, Alon Korngreen

**Affiliations:** ^1^The Leslie and Susan Gonda Multidisciplinary Brain Research Center, Bar-Ilan UniversityRamat-Gan, Israel; ^2^The Mina and Everard Goodman Faculty of Life Sciences, Bar-Ilan UniversityRamat-Gan, Israel; ^3^Department of Physics, Bar-Ilan UniversityRamat-Gan, Israel; ^4^Department of Psychology, Bar-Ilan UniversityRamat-Gan, Israel

**Keywords:** magnetic stimulation, patch-clamp, action potential, cortex

## Abstract

Although transcranial magnetic stimulation (TMS) is a popular tool for both basic research and clinical applications, its actions on nerve cells are only partially understood. We have previously predicted, using compartmental modeling, that magnetic stimulation of central nervous system neurons depolarized the soma followed by initiation of an action potential in the initial segment of the axon. The simulations also predict that neurons with low current threshold are more susceptible to magnetic stimulation. Here we tested these theoretical predictions by combining *in vitro* patch-clamp recordings from rat brain slices with magnetic stimulation and compartmental modeling. In agreement with the modeling, our recordings demonstrate the dependence of magnetic stimulation-triggered action potentials on the type and state of the neuron and its orientation within the magnetic field. Our results suggest that the observed effects of TMS are deeply rooted in the biophysical properties of single neurons in the central nervous system and provide a framework both for interpreting existing TMS data and developing new simulation-based tools and therapies.

## Introduction

TMS is a popular tool for human brain stimulation and for modulating cognitive tasks (Walsh and Pascual-Leone, 2003). A TMS coil is placed above the skull over a region of interest, for example, above the motor cortex. Passing a time variable electric current pulse through the coil generates an electromagnetic field (Polson et al., 1982; Barker et al., 1985). According to Faraday's law, this induces an electric field in the brain that stimulates cortical neurons (Walsh and Pascual-Leone, 2003). The effects of TMS are often measured by behavioral observation, for example, involuntary, brief movement of the hand following stimulation over the motor cortex (Rothwell et al., 1999). As TMS can modulate behavior, thus differing from non-invasive, passive brain imaging methods, it is a powerful tool for investigating the relation between human behavior and brain activity.

Surprisingly, while TMS has been commercially available for decades, the actions of single pulse magnetic stimulation at the cellular level have not been directly studied. Some studies have suggested that that TMS activates cortical neurons antidromically, primarily at axonal bends, bifurcations, or terminations (Amassian et al., 1992; Maccabee et al., 1993, 1998; Kamitani, 2001; Hallett, 2007). Other investigations have claimed, mostly by recording spinal volleys, that the action potential is generated more proximal to the soma (Edgley et al., 1990; Baker et al., 1995; Nielsen et al., 1995; Di Lazzaro et al., 2002; Terao and Ugawa, 2002; Pasley et al., 2009). Distal axonal activation evokes indistinguishable forward and backward information flow in the cortical network, suggesting that TMS provides a nonspecific reset signal (Walsh and Pascual-Leone, 2003). In contrast, action potential initiation at the axon's initial segment elicits the normal, forward information flow in the cortical network. We recently investigated the effects of magnetic stimulation on single neurons using compartmental modeling (Pashut et al., 2011). Contrary to published models (Roth and Basser, 1990; Basser and Roth, 1991; Basser et al., 1992; Nagarajan et al., 1993; Abdeen and Stuchly, 1994; Roth, 1994; Ravazzani et al., 1996; Ruohonen et al., 1996a; Davey and Epstein, 2000; Hsu and Durand, 2000; Kamitani, 2001; Hsu et al., 2003; Rotem and Moses, 2006; Silva et al., 2008; Salvador et al., 2011) our simulations predicted that TMS affects neurons in the central nervous system by somatic depolarization leading to initiation of actions potentials in the axon's initial segment (Pashut et al., 2011).

Driven by our theoretical predictions, we combined, for the first time, a patch-clamp setup designed for brain slice recordings with a custom-made magnetic coil. Using this novel setup magnetic stimulation was applied to acute brain slices and the response of cortical neurons recorded. Our recordings supported our theoretical prediction that the action potential was generated at the initial segment of the axon following somatic depolarization during magnetic stimulation. Interneurons and pyramidal neurons responded differently to magnetic stimulation. We show, both experimentally and computationally, that the magnetic threshold of central nervous system neurons is correlated with the size of the soma, the current threshold of the neuron, and the orientation of the magnetic coil. In combination with our previous compartmental model, the current study suggests a cellular mechanism for TMS.

## Methods

### Magnetic stimulator

**Figure 1:**
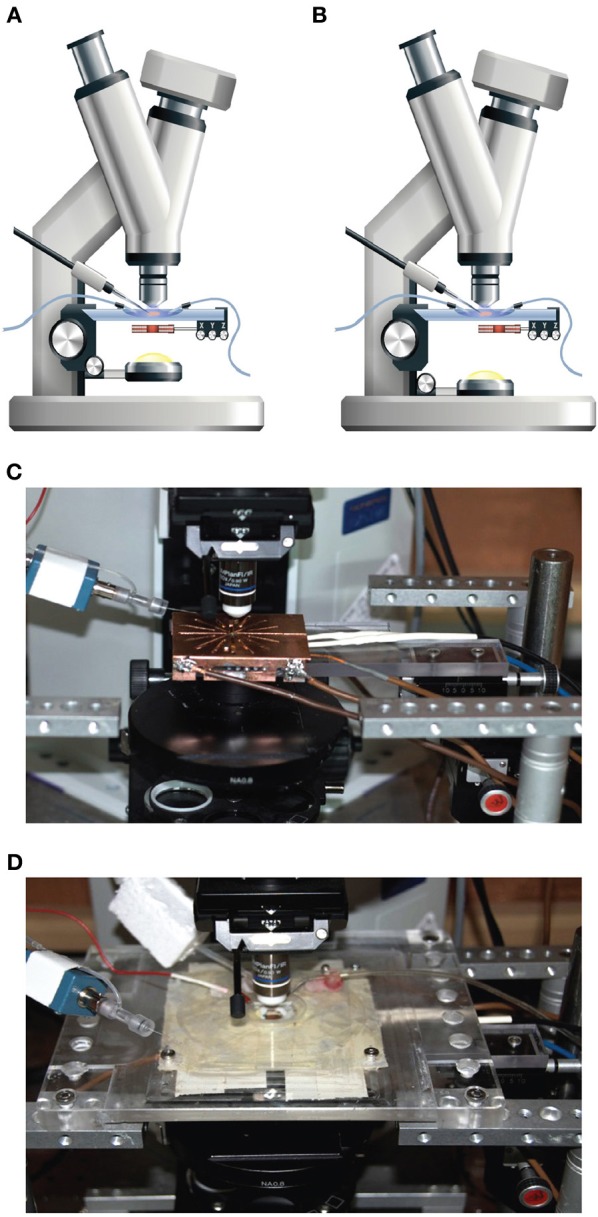
**Patch-clamp and magnetic stimulation setup. (A)** The general configuration of the modified patch-clamp setup used for patching. The coil was concentric to the light path and the condenser was elevated to allow focusing the light on the brain slice. The Z-axis distance of the coil was set at 2 mm from the plane of the slice and the Y-axis location centered with respect to the center of the coil, while the coil location in the X-axis remained flexible. **(B)** The configuration of the patch-clamp setup used for during recording. The coil was moved laterally by 1 cm to allow optimal stimulation and the condenser was lowered to reduce mechanical interactions with the coil. **(C)** An image of the setup with the specimen stage removed, allowing visualization of the shielded coil and the manual manipulator for positioning the coil. **(D)** An image of the setup with the clear plastic table with a chamber for the brain slices in the middle. The electrode headstage can be seen on the left.

A patch-clamp setup was modified to allow magnetic stimulation of cortical brain slices. Since the standard brain slice setup employs a water immersion objective it was not possible to place the magnetic coil above the brain slice. The coil was thus positioned between the condenser and the specimen table (Figure 1A). The proximity of the coil to the metal specimen table and the metal condenser induced eddy currents in these metal components, which reduced the magnetic pulse efficiency and introduced electrical noise and mechanical vibration during magnetic stimulation. To minimize the electrical artifacts we shielded the coil with a heavily grounded copper plate to reduce the radius of eddy current loops (Figure 1C). The metal stage of the microscope was replaced with a plastic one (Figure 1D), but it was not possible to replace the metal condenser. Thus, once a stable recording was established, the condenser was lowered for the duration of the experiment (Figure 1B). This greatly reduced the mechanical vibrations experienced during the magnetic pulse, except for high pulse intensities.

**Figure 2:**
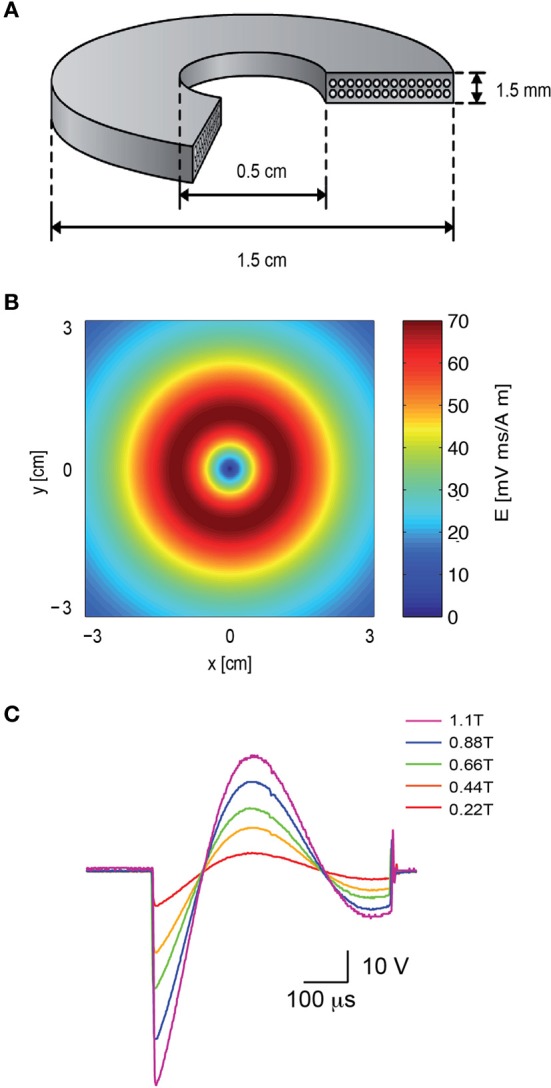
**The electromagnetic field induced by the magnetic coil. (A)** Schematic illustration of the structure of the magnetic coil. The magnetic coil was wound from copper wire of 0.75 mm diameter and constructed with two layers with 14 turns each. **(B)** The induced electric field of the coil was calculated with MATLAB, assuming a distance of 2 mm from the brain slice, and plotted along the x–y plane. **(C)** The shape and magnitude of the magnetic pulse were recorded from our coil with a pick-up coil (radius 1 cm). The signal was recorded with five different voltages applied to the capacitor bank by the high voltage power supply. The maximal magnetic field at the center of the coil is noted in color in the legend. The scale bar displays the raw voltage recorded from the pick-up coil.

To attach the patch electrode to a cortical neuron, the coil was positioned concentrically to the light path (Figure 1A). Since the induced electric field along the central axis of a round coil is zero (Figure 2B), neurons in the focal plane of the microscope are not excited when the coil is concentric to the light path. Therefore, the coil was mounted on a horizontal, plastic arm mounted on manual micromanipulator (Figure 1C). Once the patch electrode was securely connected to the neuron, the coil was moved sideways by 1 cm so that the circumference of the coil, where the induced electric field is maximal (Figure 2B), was below the neuron being recorded (Figure 1B).

A magnetic coil with the mean radius of 1 cm was forged for the magnetic stimulation of rat brain slices (Figure 2A). The design of the coil was aided using Vector Fields finite elements simulation software (Cobham Technical Services, Aurora, USA). A “wet-winding” method was used for winding a standard lacquer insulated copper wire (0.75 mm diameter). During the winding process the coil was impregnated with a low viscosity Epoxy EP29LPSP compound (Master Bond Inc., Hackensack, USA) mixed with 25 μm Alumina particles. These Alumina particles were added (at a weight ratio of 7 g Alumina to 5 g Epoxy) to reinforce the coil as well as to improve electrical insulation and heat transfer (Fridman et al., 2006). Small wire bending diameters were avoided to prevent “hot” spots of high electric fields. A high-voltage DC power supply (Model 402L,TDK-LAMBDA, Neptune, NJ, USA) was used to charge a custom-made capacitor array (200 μF).

### Simulations

The magnetic field was assessed using Vector Fields finite elements simulation software (Cobham Technical Services). The electric field induced in the plane of the brain slice was calculated using MATLAB (MATLAB 2007B, Mathworks, Natick, MA, USA) for a magnetic coil with a mean radius of 1 cm (Figure 6B), using the formulae (Tofts, 1990):

(1) $$\bar{E}=-\frac{\partial I}{\partial t}\frac{\mu_{0}N}{\pi k}{\left(\frac{r}{x}\right)}^{1/2}\left[k(m)\left(1-\frac{1}{2}k^{2}\right)-E(m)\right]\hat{\theta }$$

(2) $$m=k^{2}=\frac{4rx}{{\left(r+x\right)}^{2}+z^{2}}$$

where μ_0_ is the permeability constant, *N* is the number of loops, *I* is the current, *r* is the radius, *z* is the distance of the point from the coil plane, *x* is the distance of the point from the center of the coil, *K*(*m*) and *E*(*m*) are elliptic integrals of the first and second order and $\hat{\theta }$ is the unit vector in the direction of θ.

The changes to the membrane potential induced by the magnetic field were calculated using the activating function:

(3) $${\tilde{V}}_{m}=-\lambda^{2}\frac{\partial (E\cdot a)}{\partial a}$$

where ${\tilde{V}}_{m}$ is the change in the membrane potential generated by the magnetic stimulation, λ is the passive space constant, *E* is the induced electric field, and *a* is a unit vector parallel to the axial direction of the segment. This function, used to calculate membrane polarization due to changes in the external electric field, is known as the activating function (Rattay, 1986, 1989; Roth and Basser, 1990; Basser and Roth, 1991; Nagarajan et al., 1993; Silva et al., 2008). Equation 4 states that the strength of MS is determined by the directional derivative of the electric field along the segment direction (Silva et al., 2008) and by the intrinsic properties forming the passive space constant. From here, it is simple to derive the complete cable equation including the induced electric field (Roth and Basser, 1990; Basser and Roth, 1991; Basser et al., 1992; Nagarajan et al., 1993; Abdeen and Stuchly, 1994; Ruohonen et al., 1996b; Hsu and Durand, 2000; Rotem and Moses, 2006).

(4) $$\tau \frac{\partial V_{m}}{\partial t}+V_{m}=\lambda^{2}\frac{\partial^{2}V_{m}}{\partial a^{2}}-\lambda^{2}\frac{\partial E_{a}}{\partial a}$$

where *V_m_* is the membrane potential, τ is the time constant, *a* is the direction along the fiber and *E_a_* is the projection of the electric field in that direction.

The magnetic stimulator was simulated as an RLC circuit. All compartmental simulations were performed with NEURON 6.2 (Carnevale and Hines, 2005) using an integration time step of 1 μs (see Pashut et al., 2011 for details). Briefly, the temporal part of the electric field was calculated in NEURON in every time step. The spatial part of the electric field was calculated in Matlab prior to the simulation and exported from Matlab to NEURON with a spatial resolution of 1 μm. Neuronal excitability was simulated using a previously published model for cortical pyramidal neurons (Schaefer et al., 2003). In simulating the effect of magnetic stimulation on L5 pyramidal neurons the conductance densities and passive membrane parameters were similar to those defined in the original model (Schaefer et al., 2003). To simulate the response of low threshold interneurons we shifted the activation curve of the voltage-gated sodium channel by −8 mV. All the morphologies used in the simulations were of neurons that were recorded and stained in this study and reconstructed in Neurolucida.

### Magnetic pulse

To compare the intensity of our coil to the commercial coil a single-loop pick-up coil (radius 1 cm) was connected to an oscilloscope. The pick-up coil was centered on top of our coil while the potential across the capacitor bank was increased (Figure 2C). A bi-modal full wave cycle was generated by the system with a time constant derived from the capacitance and inductance of the system (~550 μs), longer than the ~400 μs waveform recorded using the same pick-up coil from a 2000 Super Rapid Magnetic stimulator (Magstim Company, Dyfed, UK). The peak magnetic field in each recorded sweep was measured using a 410 Hand Held Gaussmeter (Lake Shore Cryotronics, Westerville, OH) and is given in the legend for Figure 2C.

### Slice preparation

Thirteen to fifteen day old Wistar rats of either sex were killed by rapid decapitation after anesthesia with isoflurane, according to the guidelines of the Bar-Ilan University animal welfare committee. This procedure was approved by the national committee for experiments on laboratory animals at the Israeli Ministry of Health. Slices (sagittal, 300 μm thick) were prepared from the somatosensory cortex using previously described techniques (Bar-Yehuda and Korngreen, 2007; Bar-Yehuda et al., 2008). All experiments were carried out at room temperature (20–22°C). Neurons were visualized using infrared differential interference contrast (IR-DIC) videomicroscopy (Stuart et al., 1993).

### Solutions and drugs

Slices were perfused throughout the experiment with an oxygenated artificial cerebrospinal fluid (ACSF) containing: (mM) 125 NaCl, 25 NaHCO_3_, 2.5 KCl, 1.25 NaH_2_PO_4_, 1 MgCl_2_, 2 CaCl_2_, 0.499 Na-ascorbate, and 25 glucose (pH 7.4 with 5% CO_2_) or artificial cerebrospinal fluid 2 (ACSF_2_) containing: (mM) 125 NaCl, 25 NaHCO_3_, 4.5 KCl, 1.25 NaH_2_PO_4_, 1 MgCl_2_, 1.2 CaCl_2_, 0.499 Na-ascorbate, and 25 glucose (pH 7.4 with 5% CO_2_). ACSF_2_ had a higher KCl concentration (2.5–4.5) and lower CaCl_2_ concentration (2–1.2) than ACSF in order to excite neurons in the slice (Bar-Yehuda and Korngreen, 2007). In experiments where the network was to be blocked, the following blocking drugs were added to ACSF: bicuculline methiodide to block GABAa receptors (50 μM), 2-amino-5-phosphonopentanoic acid (APV) (50 μM) and 6-cyano-7-nitroquinoxaline-2,3-dione (CNQX) (15 μM) to block NMDA and AMPA receptors, respectively. The recording electrode was filled with the standard pipette solution containing (mM): 125 K-gluconate, 20 KCl, 10 HEPES, 4 MgATP, 10 Na-phosphocreatine, 0.5 EGTA, 0.3 GTP, and 0.2% biocytin (pH 7.2 with KOH). At the end of each experiment, slices were fixed in cold 100 mM phosphate buffer solution (pH 7.4) containing 4% paraformaldehyde. After fixation the slices were incubated for 2 h in avidin-biotinylated horseradish peroxidase (ABC-Elite, Vector-Laboratories, Peterborough, UK) and the stain was developed using 0.015% diaminobenzidine. The stained neurons were digitally traced using a Neurolucida system (Micro-BrightField, Williston, VT, USA) and the tracings were converted to NEURON readable code.

### Electrophysiological recordings

Recordings from neuron somata used a BVC-700A amplifier (Dagan Corp.). Voltage was filtered at 5 kHz and sampled at 10 or 40 kHz using a National Instruments analog-to-digital interface operated by procedures custom written in IgorPro 6 (WaveMetrics, Lake Oswego, USA) and stored on the hard disk of a personal computer. Patch pipettes were pulled (5–10 MΩ) from thick-walled borosilicate glass capillaries (2.0 mm outer diameter, 0.5 mm wall thickness; Hilgenberg, Malsfeld, Germany). The electrophysiological recordings were first performed in the whole-cell patch-clamp configuration followed by the magnetic threshold measurement in the loose-patch configuration.

### Analysis

Data were analyzed off-line with IgorPro 6.0 (WaveMetrics, Lake Oswego, USA) on a personal computer. Experimental results were observed in cells from two or more animals. Therefore, all the results for a particular experiment were pooled and displayed as means ±SD. Groups were compared by Student's *t*-test either paired or unpaired depending on the experiment. The type of test is indicated in the text. The squared correlation coefficient and the statistical significance of the correlation are reported for linear correlations.

## Results

**Figure 3:**
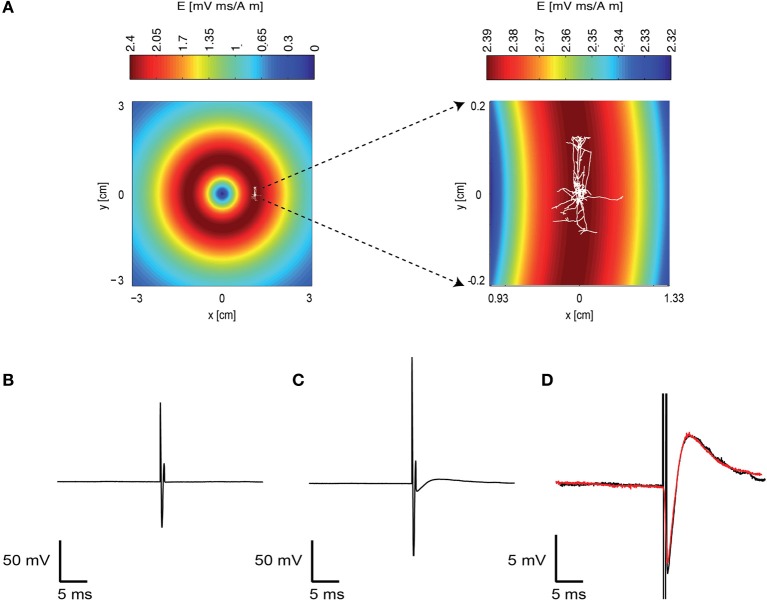
**A magnetic pulse evoked an action potential. (A)** Schematic drawing of the experimental layout. The induced electric field of the coil in the plane of the brain slice was calculated and is displayed in pseudocolor. A reconstructed L5 pyramidal neuron is overlaid on this drawing to indicate the approximate position of this neuron during the recording. The area around this neuron is enlarged on the right. Note that the induced electric field is different in the right and left panels. **(B)** A subthreshold response to the magnetic field recorded with the patch-clamp system using the loose-patch configuration. The electrode recorded the artifact caused by the magnetic stimulation. Magnetic stimulation was 0.7 T. **(C)** A suprathreshold neuron reaction to the magnetic stimulation. Magnetic stimulation was 0.9 T. **(D)** The recorded trace without the action potential **(B)** was subtracted from the trace with the action potential **(C)**. This allowed isolation of the action potential waveform (black). A spontaneous action potential is displayed in red over the action potential generated by the magnetic stimulation.

**Figure 4:**
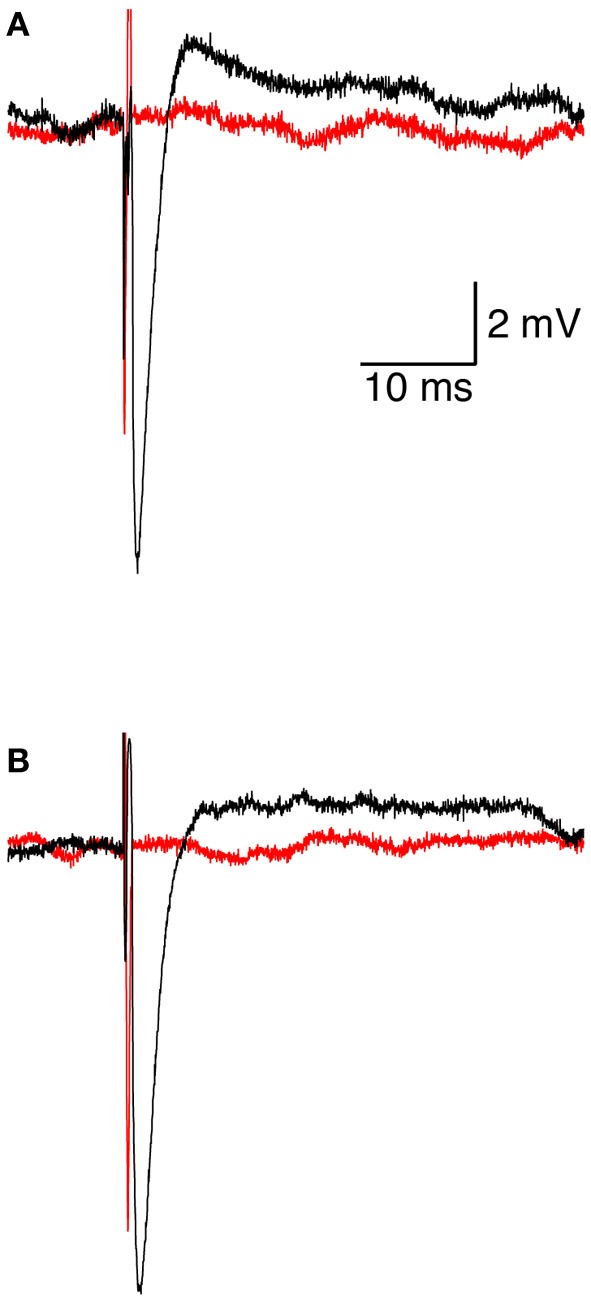
**Initiation and pharmacological block of the action potential. (A)** representative loose-patch recording of an extracellular action potential from a L5 pyramidal neuron before (black line) and after (red line) application of 100 nM tetrodotoxin. **(B)** representative loose-patch recording of an extracellular action potential from a L5 pyramidal neuron when the magnetic coil was positioned with its median radius below the neuron (black line) and when the center of the coil was positioned below the neuron (red line).

We investigated the response of a single neuron to magnetic stimulation by combining a patch-clamp setup with a magnetic coil (Figures 1, 2 in Methods). A patch electrode was attached to a layer 5 (L5) pyramidal neuron from the somatosensory cortex in the loose-patch configuration. Then, to obtain optimal stimulation, the magnetic coil was positioned with its median radius below the neuron (Figures 3A, 1A). At low stimulation intensities only a stimulus artifact was observed (Figure 3B). Increasing the intensity elicited a biphasic waveform, partially obscured by the stimulus artifact, resembling an extracellular action potential (Figure 3C). This waveform was isolated by scaling and subtracting traces recorded at low magnetic stimulation intensities from traces displaying an apparent action potential waveforms (Figure 3D). The shape of a spontaneous action potential recorded from the same neuron was identical to that triggered by magnetic stimulation (Figure 3D). Gradually increasing magnetic stimulation allowed determination of the minimal magnetic stimulation intensity required to generate an action potential. This threshold stimulation intensity is referred to as the magnetic threshold of the neuron (reported here in units of the magnetic field amplitude, Tesla, at the center of the coil). To verify that the observed waveform was indeed that of an action potential we added 100 nM tetrodotoxin to the bath solution which eliminated the action potential waveform from the loose-patch recording (Figure 4A). Similar results were obtained from three other neurons exposed to tetrodotoxin. It is well known that the induced electric field at the center of a round coil is zero and, therefore, should not stimulate action potentials. To test this we first measured the magnetic threshold of a neuron when the coil was positioned with its median radius below the neuron (Figure 4B). We then moved the coil so that the center of the coil was below that same neuron while remaining in the loose-patch configuration. As expected, the same magnetic stimulation did not induce an action potential (Figure 4B). Similar results were observed in four other neurons. This experiment verified that the induced action potential was indeed due to magnetic stimulation.

Ideally, the best configuration of the patch-clamp technique for investigating intracellular mechanisms is the whole-cell configuration (Hamill et al., 1981). However, interaction of the large electromagnetic pulse generated by the magnetic coil with the whole-cell pipette may lead to false recordings. To test this we measured the magnetic threshold of 21 L5 pyramidal neurons in the whole-cell mode and of 15 other pyramidal neurons in the loose-patch configuration. The magnetic threshold was significantly lower (0.5 ± 0.1 T, *n* = 21) in the whole-cell mode than in the loose-patch configuration (1.2 ± 0.1 T, *n* = 15, *p* < 0.0001 , unpaired *t*-test) pointing to possible interaction of the stimulus with the whole-cell pipette. We therefore performed all the recordings in this study in the loose-patch configuration. To gain access to intracellular parameters we briefly recorded the membrane potential in a current-clamp recording from each neuron in the whole-cell configuration. From these recordings we calculated the input resistance of the neuron and the current threshold of the action potential. Following this brief whole-cell recording, the patch electrode was retracted from the cell and then brought back in contact with the membrane to form a loose-patch recording configuration. In the whole-cell configuration the cytoplasm is replaced by the pipette solution. This may lead to unwanted changes in the cellular function. To rule out this possibility we recorded the magnetic threshold from several neurons in the loose-patch configuration without prior whole-cell recording. The magnetic threshold recorded under these conditions (1.3 ± 0.2 T, *n* = 5) was not significantly different than that recorded following a brief whole-cell recording (*p* = 0.63 , unpaired *t*-test).

We developed a numerical model enabling us to combine realistic magnetic stimulation with compartmental modeling of neurons with arbitrary morphology (Pashut et al., 2011). Using this model we predicted that for neurons smaller than the radius of the magnetic coil the compartment with the largest diameter (i.e., the soma) undergoes the largest depolarization. This result can be directly extracted from the activating function (Equation 3). Assuming homogenous passive parameters and a relatively shallow electric field gradient, the major difference between the soma and the other compartments in the neuron is their diameter. Since the effect of the induced electric field is scaled in Equation 3 by the passive space constant, it is largest at the soma. From this basic principle it was possible to predict that the current threshold for action potential firing would be correlated with the magnetic threshold (Pashut et al., 2011). Naturally the current threshold is a function of the input resistance, the size of the somatic compartment and the activation kinetics of the voltage-gated sodium channels responsible for action potential generation (Pashut et al., 2011). Since various classes of cortical neurons display either low or high current thresholds, we predicted that the current threshold, measured using intracellular recordings from neurons in brain slices, would be correlated with the magnetic threshold of these neurons.

**Figure 5:**
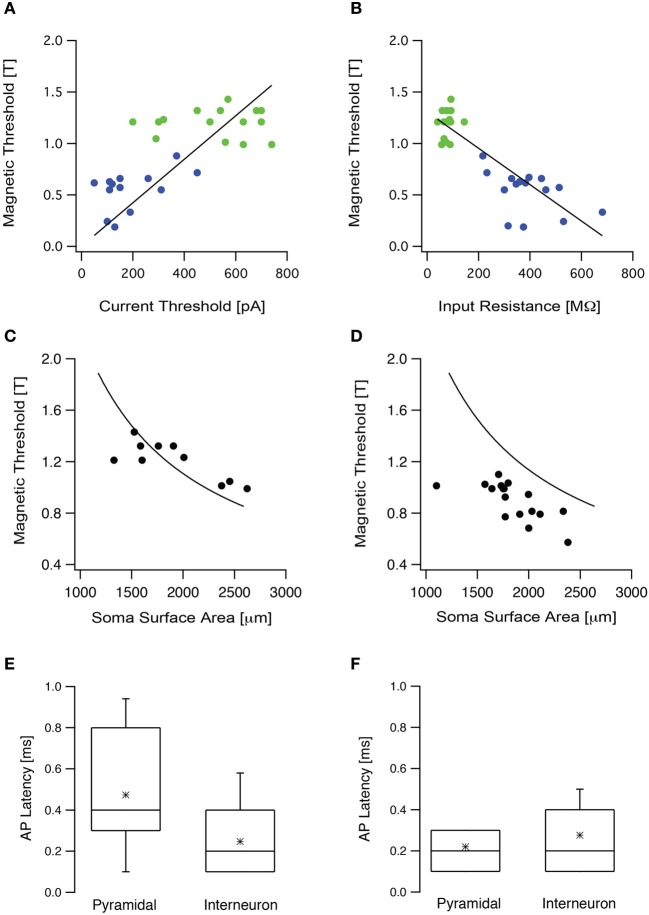
**The magnetic threshold was correlated with intrinsic cellular properties. (A)** The magnetic threshold of L5 pyramidal neurons (green circles) and low threshold interneurons (blue circles) recorded in the loose-patch configuration are plotted as a function of the current threshold recorded in the whole-cell configuration. **(B)** The magnetic thresholds of the neurons presented in **(A)** are plotted as a function of the input resistance. **(C)** The magnetic threshold recorded from L5 pyramidal neurons plotted as a function of the surface area, measured from stained neurons using Neurolucida (filled circles). The simulated magnetic threshold was calculated by systematically modifying the membrane area of a compartmental model for an L5 pyramidal neuron, while randomly modifying the surface area of the dendritic tree (line). **(D)** The magnetic threshold obtained from L5 pyramidal neurons in slices, in which the synaptic activity had been increased by replacing ACSF with ACSF_2_, plotted as a function of the surface area measured with Nerolucida from stained neurons (filled circles). The line is the same as that presented in **(C)**. **(E)** Box plot of the latency between the magnetic stimulus and the action potential recorded when the brain slice was bathed in ACSF in the presence of blockers for synaptic transmission (APV, bicuculline, CNQX). **(F)** Box plot of the latency between the magnetic stimulus and the action potential recorded when the brain slice was bathed in ACSF_2_.

To test these theoretical predictions experimentally we targeted two populations of neurons in the somatosensory cortex, L5 pyramidal neurons and low threshold interneurons. Input resistance and current threshold were recorded in the whole-cell configuration followed by magnetic threshold in the loose-patch configuration. As our simulations predicted, the current threshold displayed a statistically significant positive correlation with the magnetic threshold (*R* = 0.65, *p* < 0.05, Figure 5A), while the input resistance displayed a statistically significant negative correlation with the magnetic threshold (*R* = −0.9, *p* < 0.001, Figure 5B). In both cases there was clear clustering of the results recorded from L5 pyramidal neurons and low threshold interneurons (Figures 5A,B). We also predicted that the magnetic threshold would be correlated with the size of the somatic compartment (Pashut et al., 2011). To investigate this prediction the morphologies of a group of L5 pyramidal neurons were reconstructed using Neurolucida and the somatic surface area was calculated. The measured magnetic threshold was indeed correlated with the surface area of the somatic membrane (Figure 5C).

A third prediction from our modeling was that an increase in synaptic input would reduce magnetic threshold (Pashut et al., 2011). This prediction stems directly from the somato-centric model of neuronal excitation by magnetic stimulation. Synaptic input will depolarize the soma bringing the membrane potential closer to action potential threshold. Thus, a weaker magnetic stimulation should suffice to trigger an action potential. Increased synaptic activity in the slice can be roughly simulated in the whole-cell configuration by constant current injection. Since our experiments were limited to the loose-patch configuration we could not inject current at the soma. To induce somatic depolarization we increased synaptic activity in the slice by bathing with ACSF_2_(ACSF with increased K^+^ and reduced Ca^2+^ concentration). We have previously shown that this modified ACSF increases synaptic input to cortical neurons leading to somatic depolarization (Bar-Yehuda and Korngreen, 2007; Bar-Yehuda et al., 2008). We have reported that the average membrane potential depolarized by 5–7 mV while the membrane potential variance increased almost 10-fold from 0.03 to 0.4 mV^2^ (Bar-Yehuda and Korngreen, 2007; Bar-Yehuda et al., 2008). We have also reported that the input resistance decreased by ~10 MΩ when ACSF was replaced with ACSF_2_ and that the current threshold of the neuron decreased approximately by half from 280 to 130 pA (Bar-Yehuda and Korngreen, 2007; Bar-Yehuda et al., 2008). Thus, this manipulation could be considered as a reasonable replacement of a current injection through the whole-cell electrode. The magnetic threshold recorded under these conditions was significantly lower than that recorded in standard ACSF (Figure 5D, *p* < 0.05, unpaired *t*-test). Taken together, these experiments agree with our simulations and suggest that magnetic stimulation activates cortical neurons primarily by somatic depolarization.

Action potentials triggered following somatic depolarization are generated in the axon's initial segment of cortical pyramidal neurons (Kole et al., 2007). According to our computational prediction, magnetic stimulation induces the largest depolarization in the soma followed by action potential initiation at the axon's initial segment (Pashut et al., 2011). Proving this prediction requires simultaneous recording from the axon's initial segment and the soma. This experiment cannot be performed due to the limitation of our recording setup. Thus, we designed an experiment that provided partial verification of this prediction. The latency between the stimulus and the action potential should be short and comparable to that previously reported (Kole et al., 2007). Thus, we measured the latency between the action potential and the stimulus in L5 pyramidal neurons and in low threshold interneurons (Figure 5E). To observe only the cellular response, glutamatergic synaptic transmission was blocked with 50 μM APV and 15 μM CNQX and GABAergic synaptic transmission was blocked with 50 μM bicuculline. After blocking, the mean magnetic threshold was 1.0 ± 0.1 T (*n* = 16) for L5 pyramidal neurons and 0.6 ± 0.2 T (*n* = 9) for low threshold interneurons. The mean latency for L5 pyramidal neurons was 0.48 ± 0.24 ms (*n* = 15, Figure 5E) and for interneurons 0.25 ± 0.15 ms (*n* = 8, Figure 5E). Next, the impact of network activity on action potential latency was tested. Synaptic activity in the slice was increased by replacing ACSF with ACSF_2_ (Bar-Yehuda and Korngreen, 2007; Bar-Yehuda et al., 2008). Under these conditions the mean latency recorded for L5 pyramidal neurons was 0.22 ± 0.08 ms (*n* = 19) and for interneurons 0.28 ± 0.13 ms (*n* = 8, Figure 5F). While providing indirect proof, these short action potential latencies support our hypothesis that magnetic stimulation generates action potentials proximal to the soma, probably at the axon's initial segment or at the first node of Ranvier.

**Figure 6:**
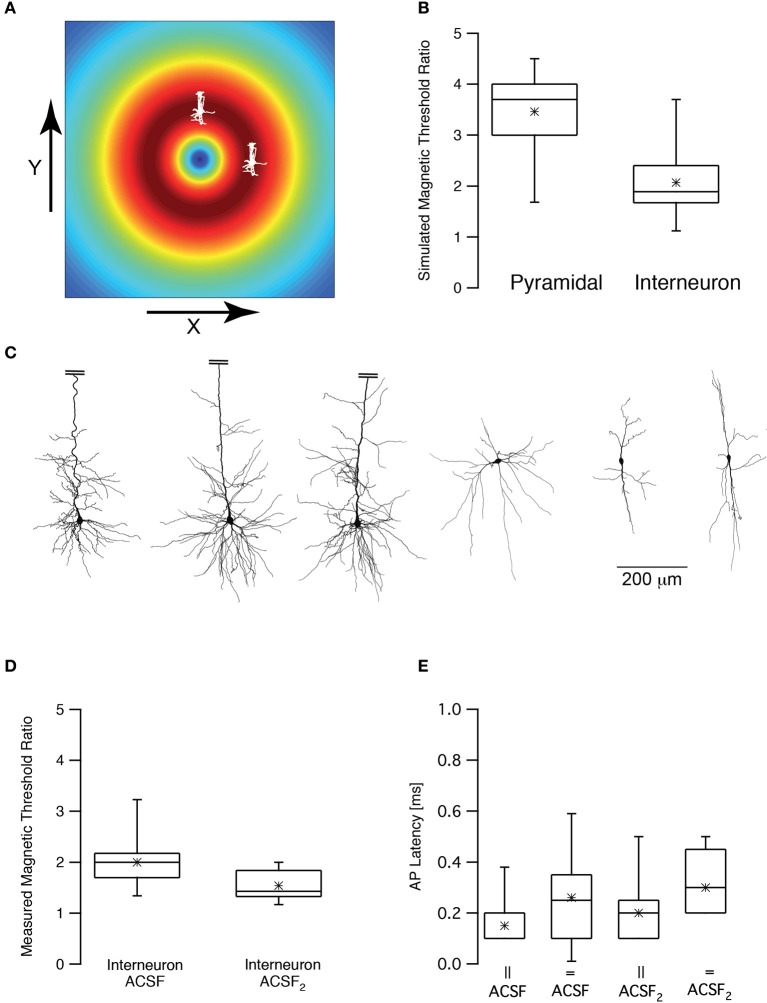
**Magnetic threshold was dependent on coil orientation. (A)** Schematic drawing of the simulated and experimental settings showing the calculated induced electric field and two pyramidal neurons, one shifted by 1 cm in the x direction and one shifted by 1 cm in the y direction from the center of the coil. **(B)** Box plot of the simulated magnetic threshold ratio. The magnetic threshold (MT) was simulated once when the neuron was shifted in the x direction and once in the y direction. The ratio was obtained by dividing the MT_x_ by MT_y_. **(C)** representative reconstructions of three L5 pyramidal neurons and three low threshold interneurons used in the simulations presented in **(B)**. The apical dendrite of the pyramidal neurons was truncated to allow using the same scale for both neuronal types. **(D)** Box plot of the measured magnetic threshold ratio recorded from low threshold interneurons. **(E)** Box plot of the latency between the magnetic stimulus and the action potential recorded during the experiments in **(C)**.

Rotation of the TMS coil above the skull can robustly change the activation of motor pathways (Day et al., 1989; Brasil-Neto et al., 1992; Sakai et al., 1997). Could we observe this effect in our numerical model and patch-clamp recordings? First, we simulated the magnetic threshold using a realistic compartmental model of cortical neurons (Schaefer et al., 2003). The magnetic threshold was simulated once when the simulated coil was shifted in the X direction by 1 cm, orienting the lines of the induced electric field parallel to the apical dendrite of a L5 pyramidal neuron (Figure 6A) and once when the simulated coil was shifted in the Y direction by 1 cm, orienting the lines of the induced electric field perpendicular to the apical dendrite of a L5 pyramidal neuron (Figure 6A). The threshold ratio (calculated by dividing the magnetic threshold simulated in the Y direction by that simulated in the X direction) of these two simulations was 3.5 ± 0.9 (*n* = 7) for pyramidal neurons. Given the average magnetic threshold recorded for L5 pyramidal neurons in our recording setup, this simulation predicted that the experimental magnetic threshold in the Y direction should be ~4 T. This was above the upper intensity limit of our magnetic stimulator. Therefore, we repeated the same simulations using morphological reconstructions of low threshold interneurons. The somata of these neurons are less elongated than those of L5 pyramidal neurons. Thus, based on our biophysical model (Pashut et al., 2011), the magnetic threshold ratio should be smaller than that calculated for pyramidal neurons. Indeed, our simulations predicted that the magnetic threshold ratio would be 2.0 ± 0.8 (*n* = 6) for low threshold interneurons (Figure 6B). It is important to note that this is a very qualitative calculation since we applied the same model used to simulate action potentials in pyramidal neurons (Schaefer et al., 2003) for the interneuron simulations. It is important to note that the difference between the simulated magnetic threshold ratios is possibly a function of several variables. Comparing the morphologies of three pyramidal neurons with those of three low threshold interneurons (Figure 6C) demonstrated the clear difference between the somatic compartments of these two neuronal types. Moreover, it was also clear that there are more dendrites emanating from the soma of pyramidal neurons than that of an interneuron. We have predicted that magnetic threshold will increase as a function of the number of dendrites connected to the soma (Pashut et al., 2011). This may contribute to the different threshold ratios we simulated.

Next we performed the experiment proposed by these simulations. We recorded the magnetic threshold from low threshold interneurons once when the coil was shifted in the X direction by 1 cm and once when the simulated coil was shifted in the Y direction by 1 cm. The experimental magnetic threshold ratio for low threshold interneurons was thus measured to be 2.0 ± 0.5 (Figure 6D, *n* = 10) agreeing with our simulations. It was possible to hypothesize, based on our theoretical predictions, that somatic depolarization will lower the magnetic threshold ratio since the membrane potential will be closer to threshold and its orientation compared to the induced electric field will be less relevant. We again induced somatic depolarization by increasing synaptic activity in the brain slice by replacing ACSF with ACSF_2_. Under these conditions the magnetic threshold ratio was 1.5 ± 0.3 (*n* = 9) for low threshold interneurons, significantly smaller than the ratio recorded in ACSF (*p* < 0.05, unpaired *t*-test). In all four experimental conditions the latencies between the magnetic stimulation and the action potential were short, supporting the hypothesis that the action potential was generated at the axon's initial segment regardless of the orientation of the magnetic coil (Figure 6E).

**Figure 7:**
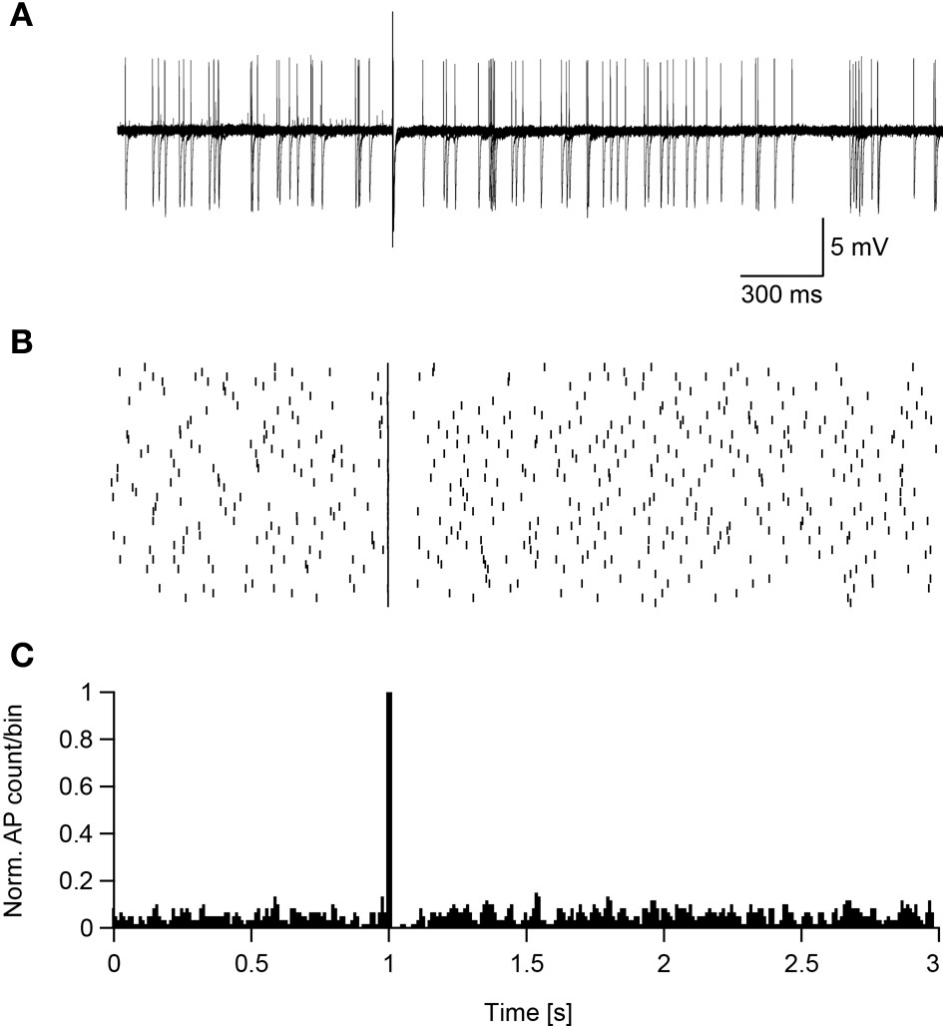
**Suprathreshold magnetic stimulation induces a pause in the spontaneous firing of cortical neurons. (A)** Ten overlaid sweeps from a low threshold interneuron firing spontaneously. Magnetic stimulation (0.24 T) was applied after 1 s of recording. **(B)** Raster plot of the neuron's reaction to the magnetic stimulus. **(C)** PSTH of the neuron's reaction to the magnetic pulse summed over 50 sweeps.

Assuming that magnetic stimulation induces action potential firing in the axon's initial segment, then suprathreshold magnetic stimulation should cause the neuron to enter a refractory period phase-locked with the stimulus. To test this hypothesis the standard ACSF was replaced with ACSF_2_, inducing an increase in the synaptic activity in the slice that caused spontaneous firing in some neurons (Bar-Yehuda and Korngreen, 2007; Bar-Yehuda et al., 2008). Such spontaneously firing low threshold interneurons were magnetically stimulated 50 times, each time inducing an action potential (Figure 7A). In all sweeps the magnetic stimulation-generated action potential was followed by a short reduction in the firing of the neuron, as shown in the raster plot (Figure 7B) and peristimulus histogram (Figure 7C). In low threshold interneurons recorded in these experiments this reduction in firing induced by the stimulus lasted 156 ± 60 ms (*n* = 13). Obviously, this pause may be the result of a combination of cellular refractory period and network activity that could not be told apart while recording in the loose-patch configuration. This experiment could not be performed with L5 pyramidal neurons; the residual mechanical vibrations at high magnetic pulse intensities did not allow collecting enough stimulation sweeps to generate a raster plot and PSTH. However, a similar pattern of activity was qualitatively observed in three L5 pyramidal neurons.

## Discussion

Here we investigated the basic mechanisms of magnetic stimulation of cortical neurons *in vitro* by combining magnetic stimulation with patch-clamp recordings in rat brain slices (Figures 1, 2). Using the loose-patch configuration of the patch-clamp technique we were able to detect action potentials following magnetic stimulation (Figures 3, 4). We presented evidence verifying the predictions of our compartmental model (Pashut et al., 2011) and supporting a mechanism in which central nervous system neurons are activated by magnetic stimulation induced somatic depolarization followed by action potential initiation in the axon's initial segment (Figures 5, 6, 7).

We modified a standard patch-clamp setup adding a custom made coil between the slice chamber and the condenser (Figure 1). It was imperative to remove as much metal as possible from the vicinity of the coil and to position a heavily grounded shield between the coil and the slice chamber (Figure 1B). Without the shield, the coil acted as one plate of a capacitor with the bath solution acting as the other plate. This generated unwanted neuronal excitation and eddy currents that were completely eliminated by the grounded shield. Indeed, when the neuron was positioned above the center of the coil it was not stimulated (Figure 4B) since the electric field induced by the magnetic pulse is zero in this location. While the electrical artifact was eliminated, we discovered that we could not record intracellular event using the whole-cell configuration. This unfortunate limitation is probably derived from the basic principle of magnetic stimulation. Since the membrane is transparent to the magnetic field it induces an electric field within the neuron generating an axial current when it interacts with the cytoplasmic resistor. Thus, a patch-pipette in the whole-cell configuration can also be viewed as a large cytoplasmic resistor contributing current to the neuron resulting in a reduction of the magnetic threshold. Since this recording artifact stems directly from the interaction of the pipette solution with the induced electric field it may well be that it will not be possible to record the membrane potential during magnetic stimulation using currently available patch-clamp amplifiers.

Similar to inducing an axial current in the solution contained within the patch pipette the induced electric field also generates an axial current when it interacts with the cytoplasmic resistor in dendrites, axons, and somata (Rattay, 1986, 1989; Roth and Basser, 1990; Basser and Roth, 1991; Nagarajan et al., 1993; Silva et al., 2008). Thus, for neurons smaller than the radius of the magnetic coil we have predicted, using numerical simulations, that the compartment with the largest diameter (i.e., the soma) will undergo the largest depolarization (Pashut et al., 2011). This result can be directly extracted from the activating function (Equation 3). Given homogenous passive parameters and a relatively shallow electric field gradient, the major difference between the soma and the other compartments in the neuron is their diameter. Since the effect of the induced electric field is scaled in Equation 3 by the passive space constant, it is largest at the soma. This somatic depolarization is attenuated by current escape into the dendrites that are less affected by the magnetic pulse due to their smaller diameter (Pashut et al., 2011). Thus, our theory predicted that the soma would experience the largest depolarization during magnetic stimulation. Consequently, the passive parameters of the somatic compartment and the excitability of the axon initial segment are predicted to determine the response of the neuron to magnetic stimulation (Pashut et al., 2011).

We tested these predictions using our experimental setup. We were able to show that, as predicted, the magnetic threshold was a function of current threshold (Figure 5A) and of the input resistance (Figure 5B). Furthermore, we attempted to verify the prediction that the magnetic threshold is correlated with the size of the soma (Figure 5C). Since we could not induce somatic depolarization directly we increased synaptic drive in the slice. This lowered the magnetic threshold, again as predicted by our numerical simulations (Pashut et al., 2011). The latencies of the action potential from the magnetic stimulus were comparable to those recorded intracellularly (Kole et al., 2007) further suggesting that the action potentials were generated at the axon's initial segment. Unfortunately, since we cannot record directly from the axon during magnetic stimulation, these results should be considered as only qualitative. We also observed that the orientation of the neuron in relation to the magnetic field is qualitatively similar between compartmental modeling and loose-patch recordings (Figure 6). Despite these limitations, the overall agreement of our results with the predictions of our numerical model support the suggestion that magnetic stimulation activates central nervous system by depolarizing the somatic compartment followed by action potential initiation in the axon's initial segment.

This suggestion is also supported by several recent experiments. For example, imaging in primary cultures of hippocampus neurons has provided some support for the relationship between magnetic threshold and intrinsic neuronal excitability; a small group of neurons responded with higher sensitivity to magnetic stimulation, promoting the concept of initiating cells in the network (Rotem and Moses, 2008). Stimulating neurons in brain slices by uniform electric fields has shown that neuronal morphology correlates with somatic subthreshold deflection of the membrane potential (Radman et al., 2009). Radman's study also observed larger somatic depolarization in L5 pyramidal neurons than in interneurons with smaller somata, fitting our predictions. Recordings of extracellular spikes and local field potential from cat cortex following TMS has clearly demonstrated that the response to TMS depends on the state of network activity (Pasley et al., 2009). Finally, recent extracellular patch-clamp recordings from retinal ganglion cells *in vitro* have shown short latency initiation of action potentials by magnetic stimulation suggesting action potential generation at the axon's initial segment (Bonmassar et al., 2012).

What is the relation between our cellular findings and the numerous results obtained when applying TMS to human subjects? Obviously, the effects of TMS on humans are complex, including a large contribution from local and distal networks (Walsh and Rushworth, 1999; Walsh and Pascual-Leone, 2003; Hallett, 2007; Pell et al., 2011). Yet, surprisingly, many of our *in vitro* results have clear correlates with TMS studies. For example, stimulation of the motor cortex with TMS generates activity that can be monitored as pyramidal tract volleys (Day et al., 1989). At low intensities, TMS generates volleys called indirect waves (I-waves). At high intensities, typically above motor threshold, TMS can trigger a direct volley (D-wave). It has been suggested that I-waves are due to the activation of low threshold neurons presynaptic to the corticospinal pyramidal neurons (Di Lazzaro et al., 2004). Agreeing with our findings, TMS below motor threshold activates inhibitory circuits in the motor cortex (Di Lazzaro et al., 1998). Moreover, voluntary hand contraction, supposedly increasing activity in the cortical network, increased the amplitude and number of I-waves following TMS (Di Lazzaro et al., 1999). Comparing our data to the recordings of corticospinal volleys is limited due to the absence of the motor threshold from our *in vitro* recordings. However, our instrument generated relatively low magnetic fields and we did not observe large network activation in the brain slice. Therefore, it is probably safe to assume that our recordings were performed below what would have been the motor threshold *in vivo*.

Using this assumption, it may be possible to hypothesize that at low TMS intensities, the somata of low threshold excitatory cortical neurons are depolarized enough to trigger action potentials in the axon's initial segment. This initial activation of local, low threshold, cortical networks may then drive deep pyramidal neurons to fire that may culminate in I-waves. The pause in the firing we observe following magnetic stimulation (Figure 7), occurring simultaneously in many neurons, may form the basis of I-wave synchronization and timing. As the intensity of TMS increases, more neurons are recruited, leading to the appearance of more I-waves in the pyramidal tract volley possibly reaching motor threshold. Moreover, the effects of TMS depend on the level of activity in the network (Silvanto et al., 2007a,b, 2008). Here we showed that increasing the activity of the network in an acute brain slice reduced action potential threshold during magnetic stimulation (Figure 5). This biophysical finding highlights the suggestion that more care should be taken to monitor and control the state of the subject during a TMS session to reduce variability.

In conclusion, the convergence of our cellular study with behavioral data in humans strongly suggests that the effect of TMS is correlated with the cell type and network state. This may explain, in part, the considerable variability observed between and within many brain stimulation studies. Moreover, our work demonstrates impressive correlation between the biophysical properties of single cortical neurons and results obtained when applying TMS to humans and lab animals. Thus, it is possible to suggest a conceptual model in which a single pulse of TMS activates a large population of somata in the cortex depending on their biophysical properties and their level of synaptic input at the moment of the pulse. The almost immediate firing of these neurons followed by a refractory period perturbs the cortical network, perhaps initiating the process termed “virtual lesion” (Pascual-Leone et al., 2000) and resetting the stimulated region, while the cortical network conveys the perturbation to more distal targets.

### Conflict of interest statement

The authors declare that the research was conducted in the absence of any commercial or financial relationships that could be construed as a potential conflict of interest.
